# How Cancer Research UK is adapting to adaptive designs

**DOI:** 10.1186/1745-6215-12-S1-A6

**Published:** 2011-12-13

**Authors:** Julie Hearn, Nicola Keat, Kate Law, Rowena Sharpe

**Affiliations:** 1 Cancer Research UK Clinical Trials Team, UK

## 

Clinical trials are central to Cancer Research UK’s purpose of carrying out world-class research to improve our understanding of how to prevent, diagnose and treat cancer.

The UK leads the world in the percentage of cancer patients entering clinical trials. In 2009, this figure reached an all time high of 16.8% of NHS cancer patients, of which ¾ entered a Cancer Research UK (CR-UK) supported trial (n=31,000 patients). Consequently, CR-UK has a major impact on the availability of treatment trials for UK cancer patients.

The strengths and limitations of randomised controlled trials have been discussed at length in the medical literature. In his 2008 Harveian Oration entitled *‘On the evidence for decisions about the use of therapeutic interventions’*, Professor Sir Michael Rawlins made a plea for investigators to continue to develop and improve their methodologies and for decision makers to avoid adopting entrenched positions about the nature of evidence. The clinical trials community has responded by proposing innovative trial designs and funders are considering how to respond to the challenges of funding trials with novel designs.

Multi-arm, multi-stage trial designs, for example, can increase the chance of a single trial providing a positive results and saves time and potentially money compared to separate sequential trials. CR-UK already funds several trials of this design, including STAMPEDE in prostate cancer and ICON 6 in ovarian cancer. We also fund trial the AML16 trial in Acute Myeloid Leukaemia which adopts a complex design to evaluate a number of agents concurrently dependent on the characteristics of patient sub-groups and their response to treatment. The RATH-L trial in Non-Hodgkin’s Lymphoma adopts another innovative design, evaluating both the use of PET scans to determine treatment pathways and sub-randomisations to less or more intensive treatments. Trials such as these present issues that funders need to adapt to, including (1) educating our funding committees, (2) developing processes for the continual assessment of treatments being discarded or introduced, and (3) consideration of optimal models. The measures CR-UK has taken to adopt an inclusive approach to innovative trial designs will be described.

